# Assessing the Impact of the Novel Sperm Selection Technique 'Annexin-V Coated Polystyrene Bead Technique' on Mouse Assisted Reproductive Techniques Outcomes: Preliminary Findings

**DOI:** 10.1007/s43032-024-01620-w

**Published:** 2024-06-14

**Authors:** Seda Karabulut, İrem Yalım Camcı, Ceren Erdem Altun, Melek Usta, Pakize Yiğit

**Affiliations:** 1 Department of Histology and Embryology, Istanbul Medipol University, International School of Medicine, Beykoz, Istanbul, Türkiye; 2 Health Science and Technologies Research Institute (SABİTA), Beykoz, Istanbul, Türkiye; 3 Department of Molecular Biology and Genetics, Gebze Technical University, Kocaeli, Türkiye; 4 Department of Biostatistics and Medical Informatics, Istanbul Medipol University, School of Medicine, Beykoz, Istanbul, Türkiye

**Keywords:** Sperm selection, ICSI, Infertility, APB-Tech, Embryo

## Abstract

ICSI is one of the most commonly used techniques to treat infertility. The sperm selection for the procedure is done ‘randomly’ by the embryologist according to the motility and morphology parameters which is known not to reflect the potential of a sperm for fertilization, pregnancy and a healthy childbearing. Since the apoptosis rate is higher in sperm cells of infertile patients, it is more likely to choose an apoptotic sperm by the 'random selection method'. We recently introduced a novel sperm selection technique namely ‘Annexin-V coated polystrene bead technique’(APB-Tech), for the selection of non-apoptotic sperm cells. The principal of the technique is based on the binding affinity of an apoptotic sperm to ‘Annexin-V covered beads’ enabling to distinguish a viable and a healthy sperm by light microscopy. The aim of the present study was to observe the effects of this technique on ICSI outcomes in mice. Sibling-oocyte trial was conducted and the outcome measures were compared with the results of traditional sperm selection method. Embryo and blastocyst qualities and blastocyst development rates were significantly increased in APB-Tech group, while the other parameters were not affected. Promising results obtained from the technique reflect its promising potential as a new and powerful tool for sperm selection and thus infertility techniques.

## Introduction

Infertility is a worlwide significantly increasing health problem affecting more than 15% of the couples planning to conceive [1]. Intracytoplasmic sperm injection (ICSI) is the most frequently used assisted reproduction technique accounting for 70-80% of the cycles [2] although the success rate cannot exceed 40% [3]. During natural conception, the healthiest sperm and oocyte are naturally selected to obtain the healtiest offspring. However, if the pregnancy is achieved via ICSI, the offspring may face certain health risks possibly as a result of bypassing these mechanisms. A recent meta-analysis reported an increased overall risk of chromosomal abnormalities in ICSI compared to both standard IVF (OR 1.42 (95% CI 1.09-1.85)) and natural conception (OR 2.46 (95% CI 1.52-3.99)) and an increased risk of de novo anomalies in ICSI compared to natural conception (OR 2.62 (95% CI 2.07-3.31)) in the newborn [4].

Apoptosis is an important physiological process that eliminate damaged or abnormal cells of body including the gametes [5]. Approximately 75% of the spermatogonia die via apoptosis during spermatogenesis [6, 7]. Sperm cells are more prone to DNA damage because of their limited repairing capacity [8, 9] which trigger apoptosis to prevent the offspring. As a result, DNA damages are observed more frequently in sperm cells than in oocytes [10]. However, if apoptosis is triggered in a cell, no visible and detectable markers are represented until the end of the process,which makes it impossible to visually recognise or distinguish [11].

Since the fate of ICSI is directly correlated with the quality of gametes among many other contributors, it is of great importance to select most compotent gametes without any type of abnormality [12]. However, gamete selection can only be applied to sperm cells because of the limited number of oocytes (~15) [13].

The most common sperm selection methods are based on choosing the "best looking sperm" randomly by the embryologist with the best morphology and motility which is not enough to reflect the potential of a sperm for healthy fertilization, pregnancy and childbearing [14]. Besides, since sperm DNA fragmentation rate is known to be higher in infertile patients [12], it is more likely to choose an apoptotic sperm with fragmented DNA by this selection method. A meta-analysis reported a 7.3 times higher pregnancy rate when sperm DNA fragmentation index (DFI) is less than 30% [15]. In addition, sperm freezing, which is a commonly used auxiliary method to preserve fertility, is known to cause apoptosis and DNA breaks due to the cryodamage [16, 17].

In the light of these data, it is of great importance to be able to choose the healtiest sperm, which is more likely to have the highest potential for a healthy pregnancy and offspring during ICSI.

Various techniques such as swim-up (SU), density gradient seperation (DGS), microfluidics [18], magnetic cell separation (MACS) [19], ultra-high magnification (IMSI) [20], hyaluronic acid binding [21], electrophoresis [22] and artificial intelligence applications [23] are available for sperm selection in the IVF laboratory, which have various advantages and disadvantages over each other. Most of these techniques only consider morphology and motility parameters of sperm, ignoring the DNA fragmentation parameter [24], which is associated with embryo quality and miscarriage rates [14]. Among these, the most commonly used techniques are SU and DGS [25], which are reported not to provide a sperm population with an acceptable level of intact DNA [26–28]. Hyaluronan binding assay tests the maturity of the sperm cells [21] while microfluidics applications [18] and artificial intelligence [23] evaluate the morphology and motility parameters. Among a few technique developed for apoptotic sperm selection [19, 22], none of them could find place in IVF labs because of their need for extra and expensive equipments, training, and high costs. In all of these techniques, a sperm population that is more likely to be healthy is obtained and sperm selection is done randomly from this population and none of them ensure to select a ‘single’ and a ‘viable’ sperm cell for microinjection.

Recently, our group developed a novel technique to eliminate apoptotic sperm cells namely ‘Annexin-V coated polystrene bead technique’(APB-Tech) which is patented by Turkish patent and trademark institute with an access number of E-39616753-110-220554024 (28.07.2022).

The technique’s principal is based on the binding ability of Annexin-V covered polystrene beads to the phosphatidylserine that is externalized at the outer leaflet of an apoptotic sperm's plasma membrane. The technique is optimized, and tested for validity and toxicity which were reported previously [29].

The aim of the present study was to analyse the effects of this novel technique on ICSI outcomes in mice. It will be faving the pathway for human use and thus will help to increase the outcome parameters and success rates of ICSI.

## Materials and Methods

### Ethical Statement

The present study was conducted in accordance with the ethical standards of the Declaration of Helsinki and national guidelines. The Ethics Committee of Istanbul Medipol University (38828770e604.01.01 E.55888, October 9, 2019) approved the study.

### Study Design

A randomized sibling-oocyte trial was conducted in order to evaluate the effect of APB-Tech solution when compared with traditionally selected spermatozoa. Randomization was performed during oocyte pick-up and the cumulus oocyte complexes (COCs) were collected to different dishes. Oocyte denudation were performed with hyaluronidase and pipetting.

### Animals

12 female and 12 male BALB/c mice obtained from Medipol University Experimental Animal Research and Application Center (MEDİTAM), were used for the study. Ovarian stimulation were applied to female mice and sperm retrieval to male mice. All mice were kept in an environment with a temperature of 22 ± 2 °C, a room humidity of 50 ± 2% and a light/dark cycle of 12/12 h and were fed Ad libitum with a standard diet. Weight of all mice were recorded on the first day of the study. Female mice who gained ≥ 10% weight, were discarded in order to eliminate mice with ovarian hyperstimulation syndrome (OHSS).

#### Sperm Retrieval

**Fig. 1:**
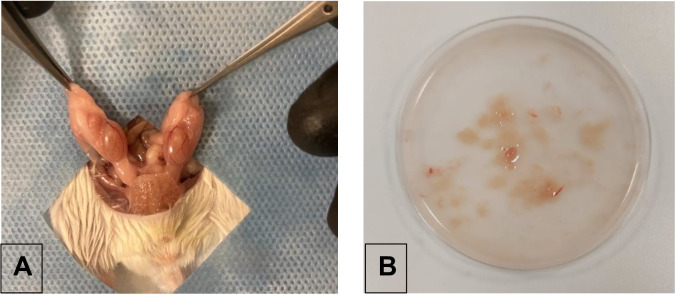
Sperm retrieval procedures are demonstrated **A**. Testis removal **B**. Testicular tissue cut into pieces are demonstrated

Sperm retrieval were performed from male mice as previosly described [29]. Mice were euthanized by CO_2_ inhalation. After cleaning the genital area with 70 % alcohol, a scrotal incision of 1 cm is cut via a lancet followed by the testis removal (Fig. 1A). Testicles were placed in a culture dish filled with pre-warmed (37°C) HEPES buffered G-Gamete (Vitrolife, Sweden) (60 mm , Falcon 353004, BD, USA) and cut into pieces mechanically by an insulin syringe (Fig. 1B). All surgical procedures were performed under sterile conditions. The tissue extracts were collected in a conical based test tube (Falcon 352095, BD, USA), centrifuged for 10 min (300 G), and suspended in 2 ml of the same media. Sperm parameters were evaluated by light microscopy (Nikon E400 ; Niko n Instrument Group, USA) and samples were kept in 37 °C incubator ( J.T.Baker, 7722-84-1, Thermo Fisher Scientific, Oslo, Norway) until use.

### Preparation of APB solution

APB solution is prepared as previously described [29]. Briefly, 200 μl (1 mg) of bead suspension, (streptavidin polystyrene, 3.11 μm, Spherotec Inc, USA) were washed according to the manufacturer's instructions. The solution is suspended in 400 μl of binding buffer (PBS + % 0.05 BSA + CaCl2, pH 7.4) and centrifuged at 1.5K for 20 min X 4. Five μg biotinylated Annexin-V (Annexin A5, Sino Biological Inc. Eschborn, Germany), per mg of beads is mixed with beads and incubated at room temperature for 30 min. The solution is then centrifuged and washed four times in PBS/BSA and is resuspended with PBS/ BSA. The solution is stored at 4 °C.

### Sperm Preperation and Selection

**Fig. 2:**
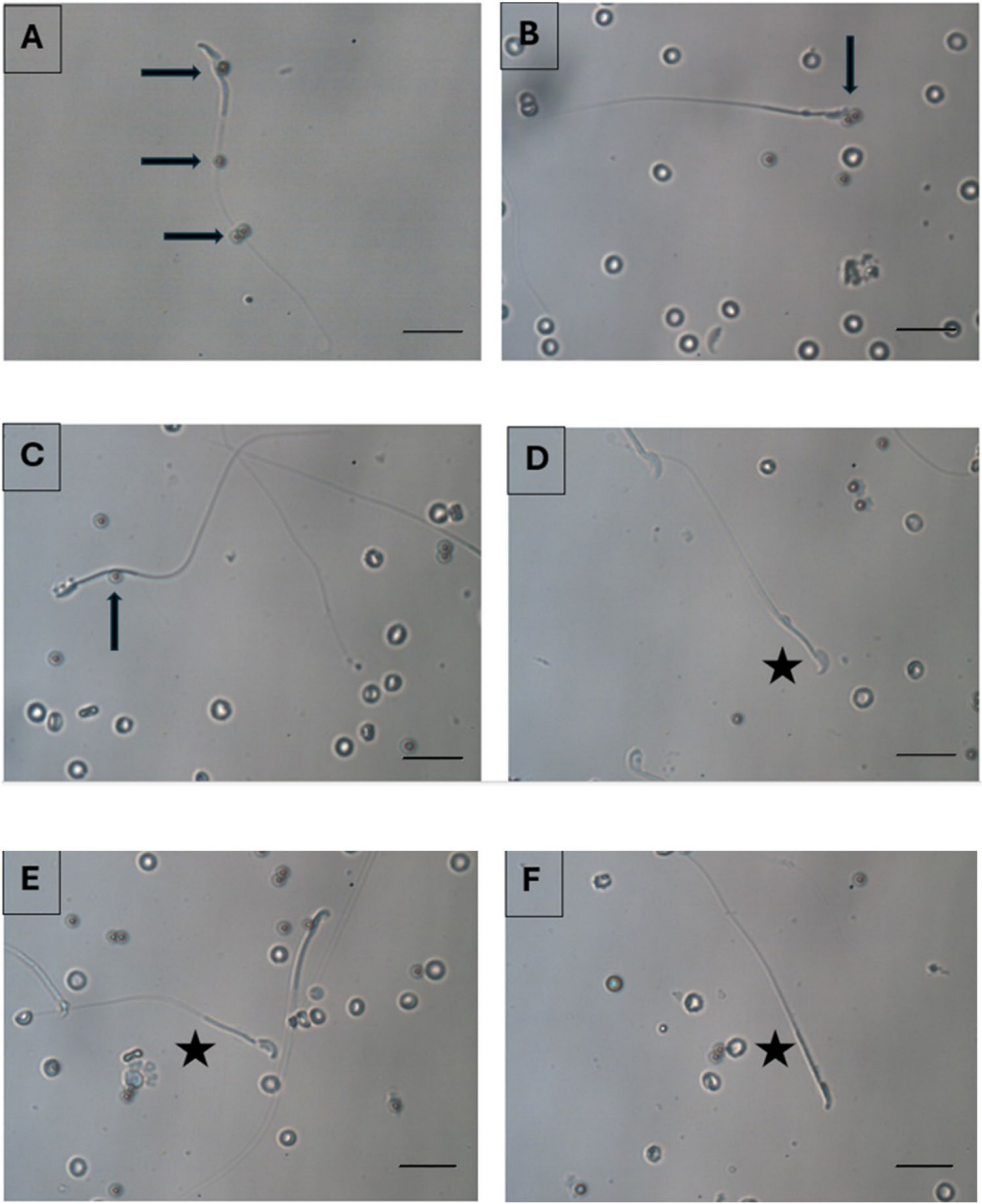
Pictures after the sperm suspension is exposed to the APB solution including the beads. Arrows (➨) show bead bounded sperm cells with DNA fragmentation, asterix (★) show unbound sperm cells with intact DNA (X40)

**Fig. 3:**
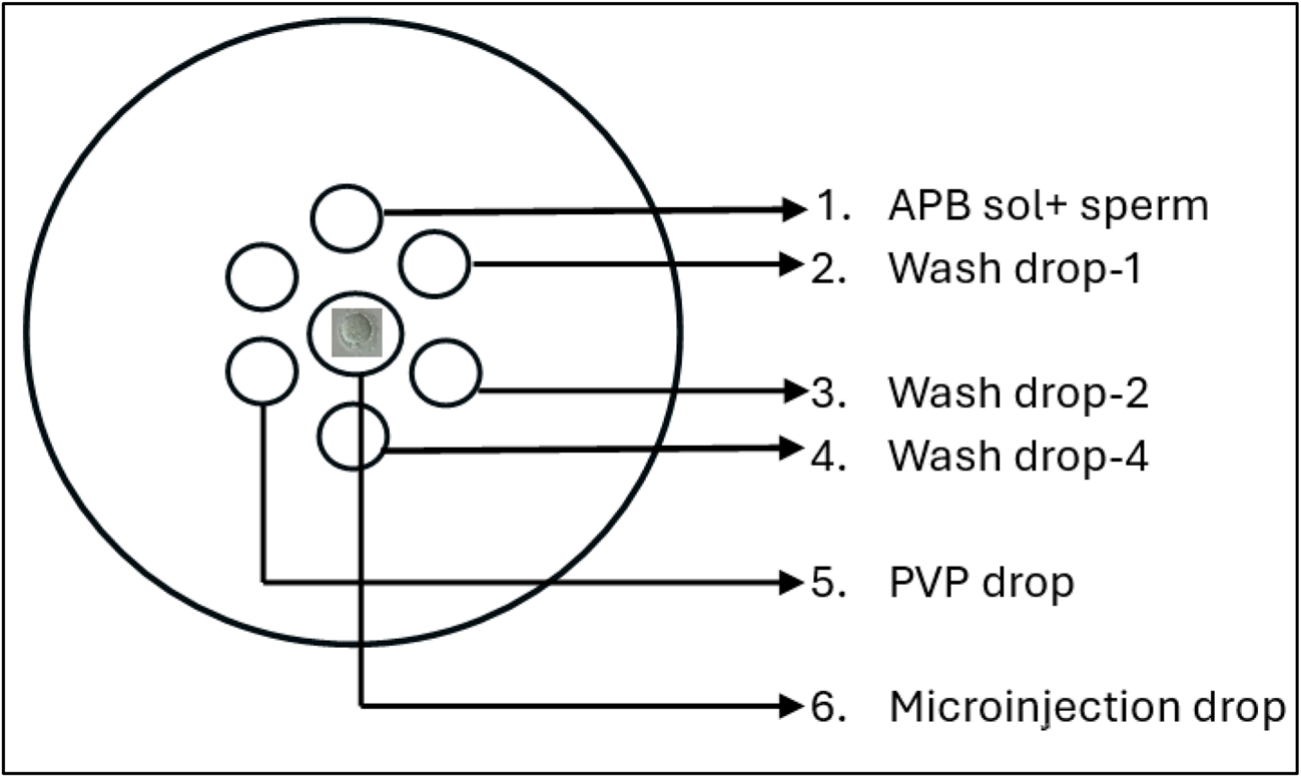
Sperm selection steps via APB-tech were demonstrated on a microinjection dish for the microinjection (ICSI) procedure

Neat semen were first preparaed by density gradient centrifugation to eliminate dead, non-motile sperm and seminal plasma as described previously [29]. Shortly, after washing, sperm concentration was adjusted to 5 ml/mL. For the study group, 10 μl of sperm solution was mixed with 10 μl of annexin-V coated bead solution (APB sol) and incubated for 30 minutes at 37°C heated stage. After the incubation, sperm cells with no bead attached on any part of them were collected with a microinjection needle. The bound and unbound sperm images are demonstrated in Fig. 2. The sperm were washed sequentially 3 times in different medium drops (10 μl) and finally transferred to a 5 μl of polyvinylpolypyrrolidone (PVP) drop to be immobilised. For the control group, 3 μl of sperm sample is added onto a 5 μl drop of PVP (ICSI, Vitrolife) solution for immobilisation in a microinjection dish (Falcon 1006, BD, New York, USA) (Fig. 3).

### Ovarian Stimulation and Oocyte Collection

**Fig. 4:**
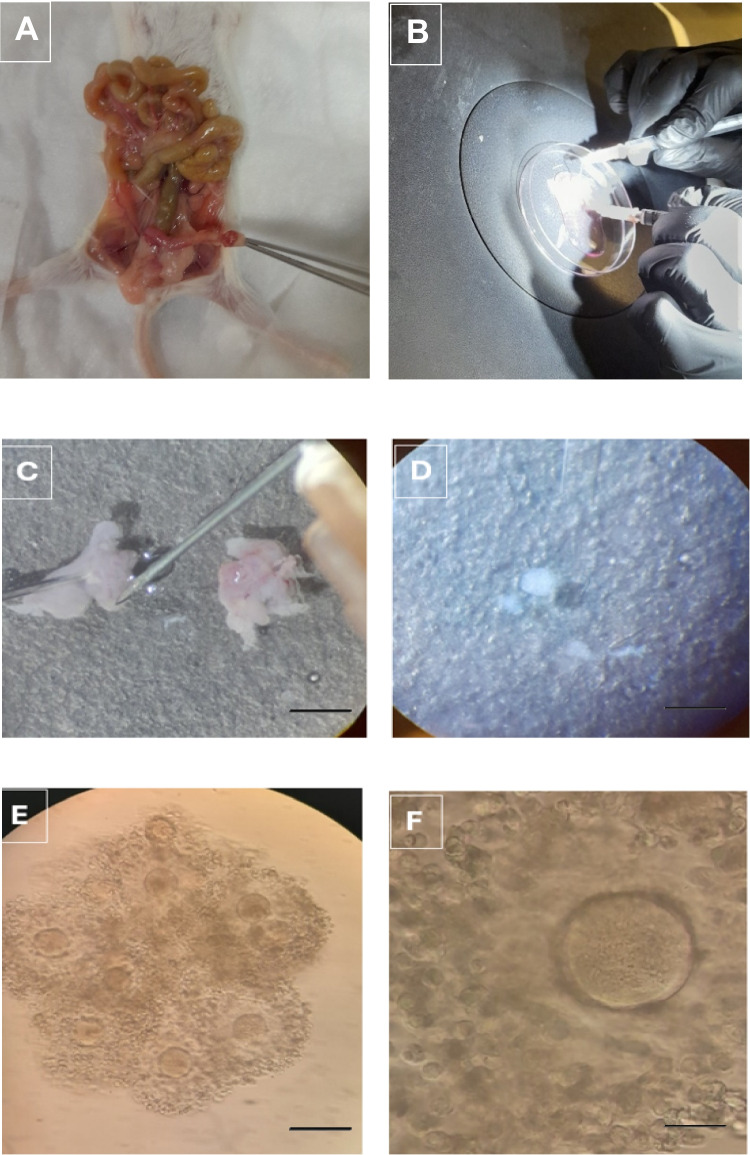
The ovary and Fallopian tubes are demonstrated after mouse decapitation (**A**). Ovary and Fallopian tubes are isolated and dissected under a stereo microscope (**B**). Ampulla region (**C**) and the contents of the ampulla shed after the rupture of the ampulla region (**D**). Cumulus-oocyte complexes (COC) are demonstrated (**E**). Photographs of an oocyte cell surrounded with corona radiata cells are demonstrated (X40)

Female mice were given subcutaneous injections of PMSG (10 IU) on 2 consecutive days, following an 30 IU of hCG administration on day 3 to induce ovulation. Mice were sacrificed 26 h post-hCG injection for oocyte collection. The ovaries and fallopian tubes were removed and washed with prewarmed G-MOPS media (G-MOPS, Vitrolife, Sweden). Ampulla regions of both side were isolated and the oocytes were collected by making a small incision in the ampulla (Fig. 4A,B,C,D) and by dissecting the ovaries under a stereo microscope. Retrieved oocytes were collected in a prewarmed (37°C) MOPS buffered media (G-MOPS, Vitrolife, Sweden) under mineral oil (Ovoil, Vitrolife), (Fig. 4E and F).

**Fig. 5:**
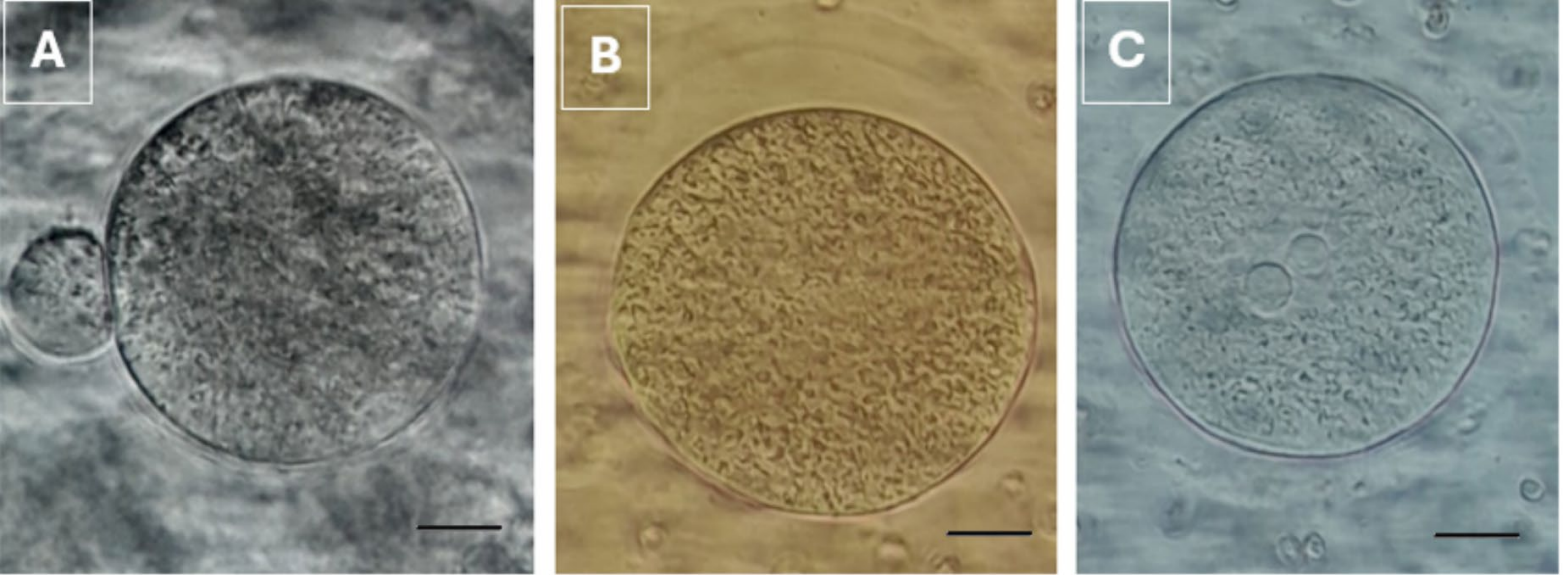
Oocyte cells after denudation are demonstrated at different stages of maturity. A mature oocyte in the Metaphase 2 (MII stage) (Polar Body is shown by the arrow) (**A**), an immature (immature) oocyte in the Metaphase 1 (MI stage) where the polar body is not observed (**B**), and an immature oocyte in the germinal vesicle (GV stage) shown by the arrow (X40) (**C**)

Two hours after the oocyte pickup (OPU) procedure, the oocytes were denuded by pipetting the COCs in 40 IU hyaluronidase (HYASE, Vitrolife, Sweden) via 2 different sized denudation pipettes (170–140 μm resp.). Oocytes were then washed 3 times in G-MOPS and placed in a culture dish containing 25 μl of G-IVF drops (Vitrolife, Sweden). The culture dishes were put in a CO_2_, O_2_, N_2_ controlled and humidified incubator for culturing. Nuclear maturation of the oocytes was assessed with an inverted microscope (X40). Oocytes with a polar body (PB) and oocytes that extrude their polar body within 4 h after denudation were defined as a mature oocyte. Oocytes with no polar body or with a germinal vesicle were defined as immature oocytes (Fig. 5) [30].

### Microinjection (ICSI)

**Fig. 6:**
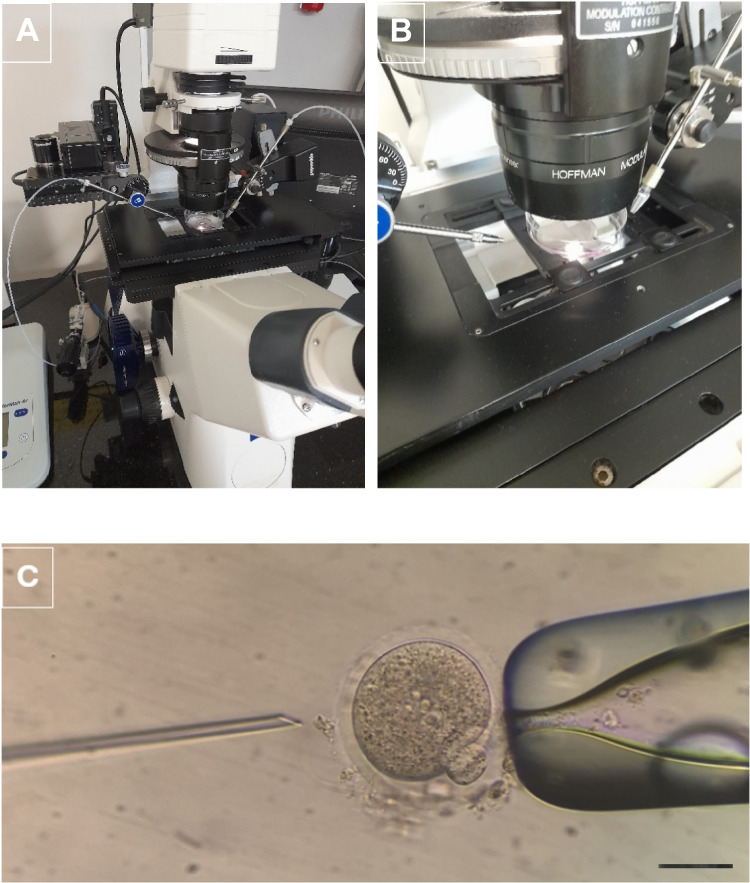
The micromanipulator that the microinjection process is performed (**A**), the dish in which the oocytes are placed during the microinjection process (**B**), and the microinjection process (**C**) are demonstrated (X20)

Mature oocytes were microinjected with a sperm as described previously by Van Steirteghem et al [31]. Shortly, after adjusting the polar body location to clock 12:00 position, and stabilizing the oocyte by aspirating with a holding pipette, an immobilised sperm is injected to the oocyte cytoplasm with a spiked microinjection pipette, a piece of cytoplasm is absorbed and the sperm together with the cytoplasm is released back to the oocyte cytoplasm (Fig. 6).

### Embryo Culture and Evaluation

**Fig. 7:**
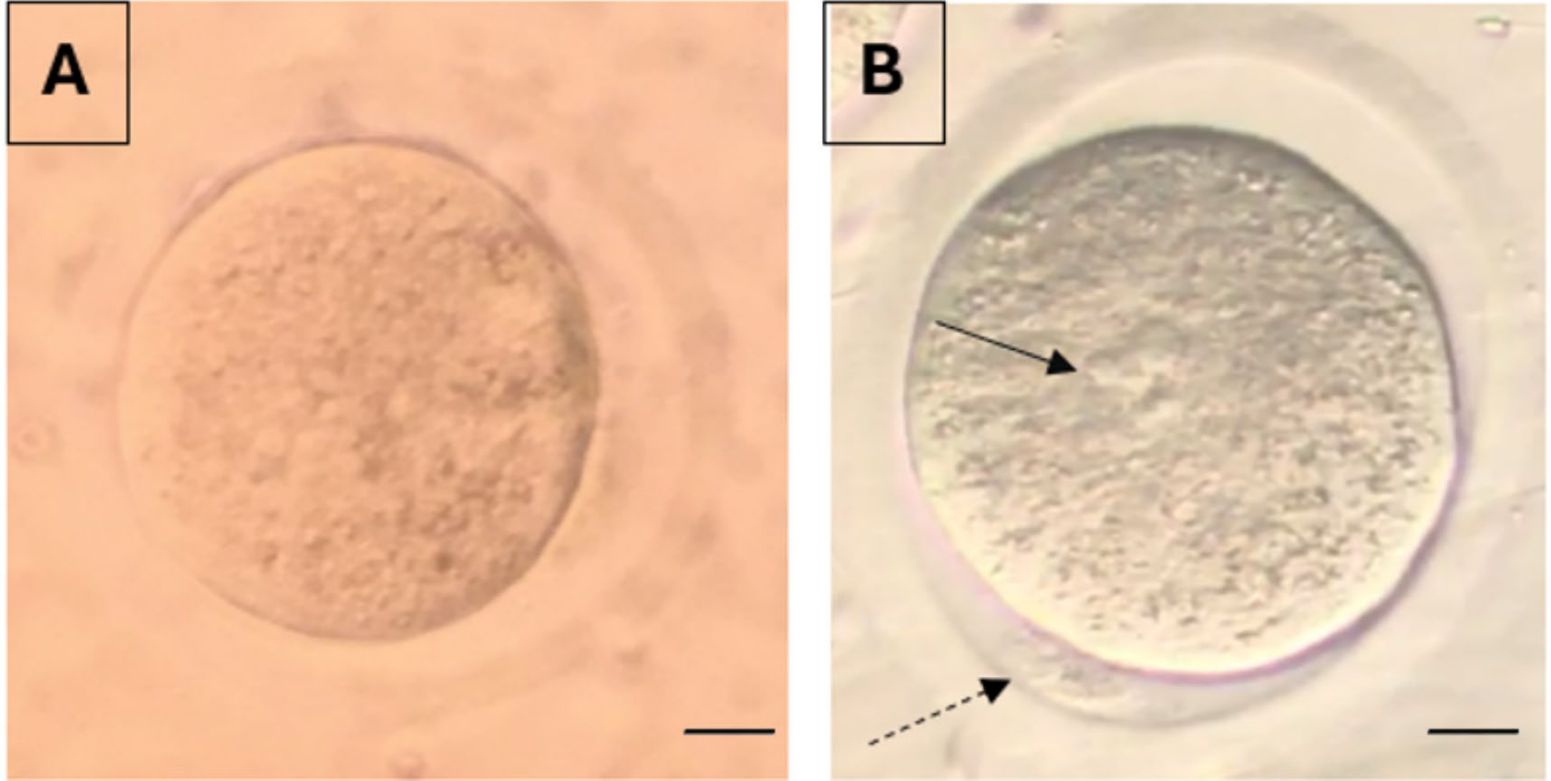
A non-fertilized (**A**) and a fertilized oocyte (**B**) with 2 pronuclei (arrows) and 2 polar bodies (dashed arrows) are demonstrated (X40)

**Fig. 8:**
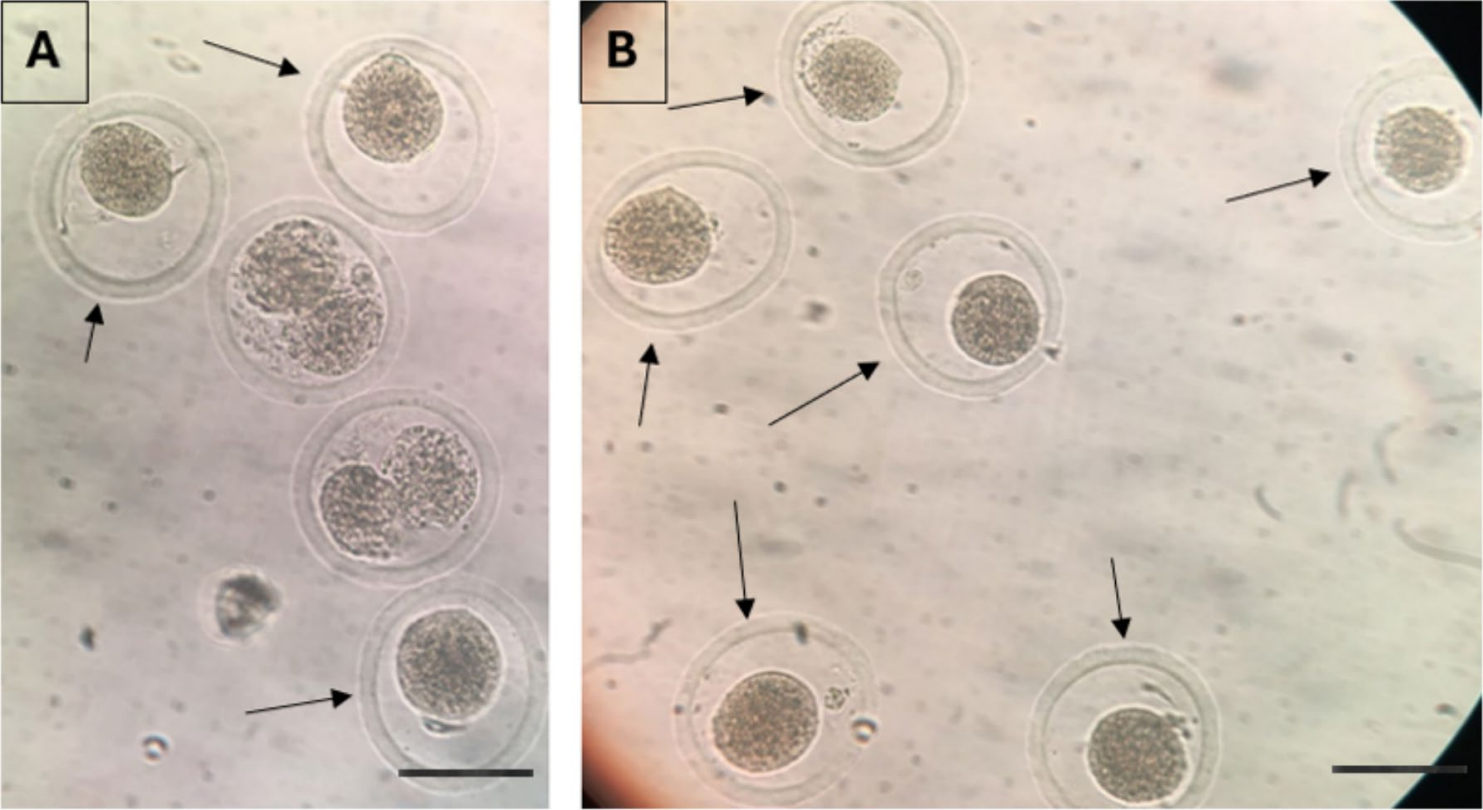
Degenerated oocytes (**A**, **B**) (cytoplasms are dark colored and retracted) are demonstrated (X20)

Further culture of injected oocytes was performed in 25 mL microdrops of culture medium (IVF, Vitrolife, Sweden) under light paraffin oil (OVOIL, Vitrolife, Sweden). All embryos were cultured for 5 days in a 37°C humidified incubator. Fertilization was evaluated 18 h after microinjection. Presence of two pronucleus and two PBs were accepted as signs of normal fertilization (Fig. 7). Fertilization rates were calculated as the number of fertilized oocytes / the number of mature oocytes ×100. Oocytes showing signs of degeneration (dark colour, shrinkaged cytoplasm) were considered as degenerated oocytes (Fig. 8).

**Fig. 9:**
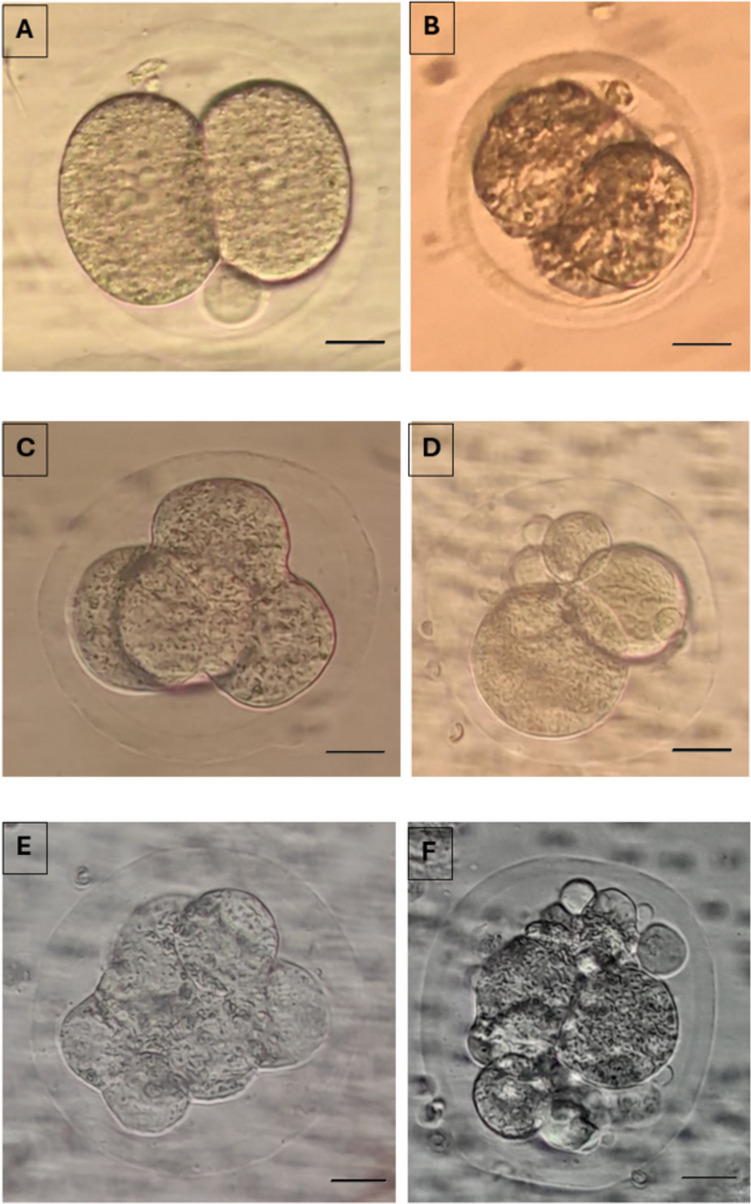

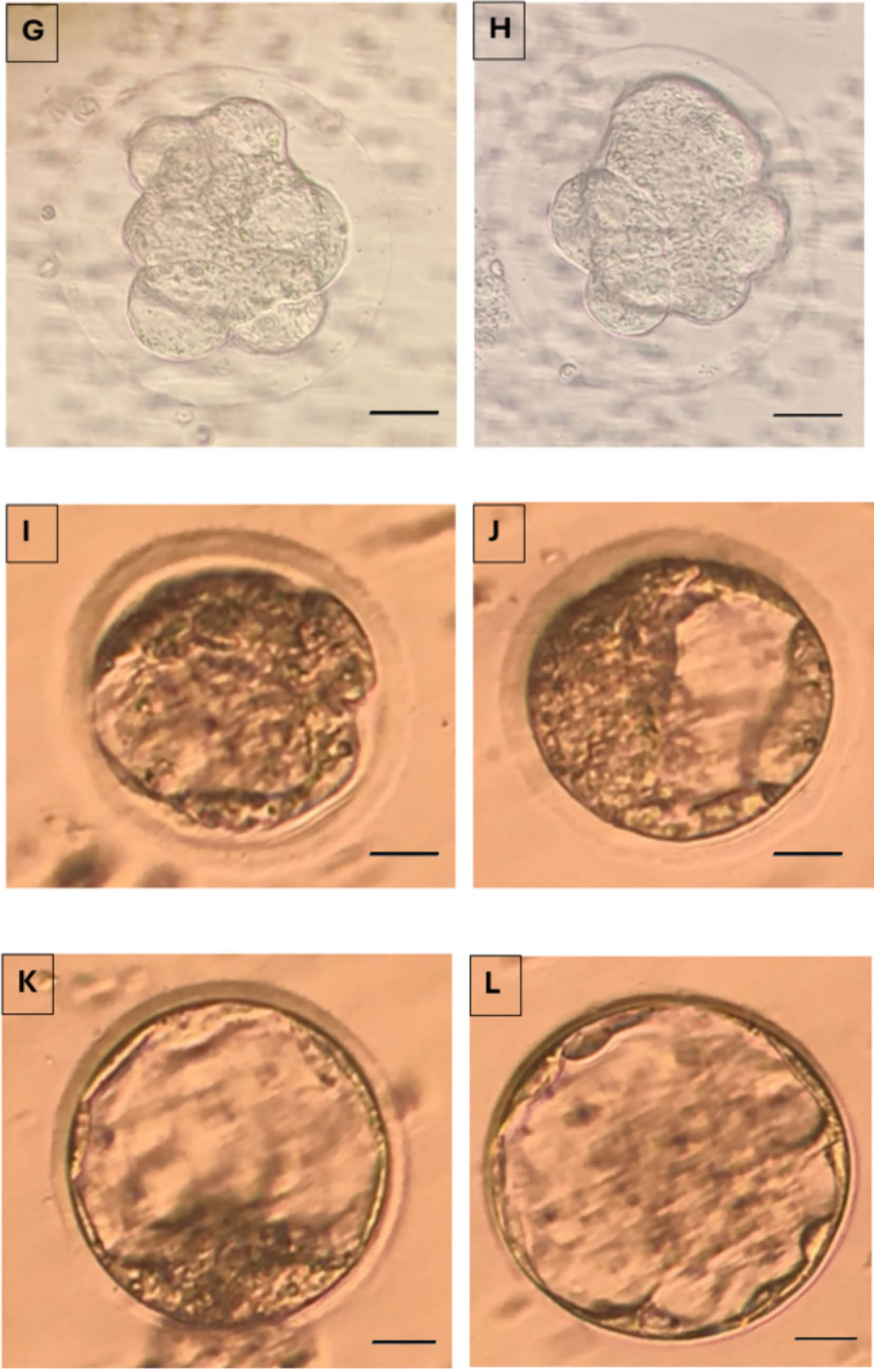

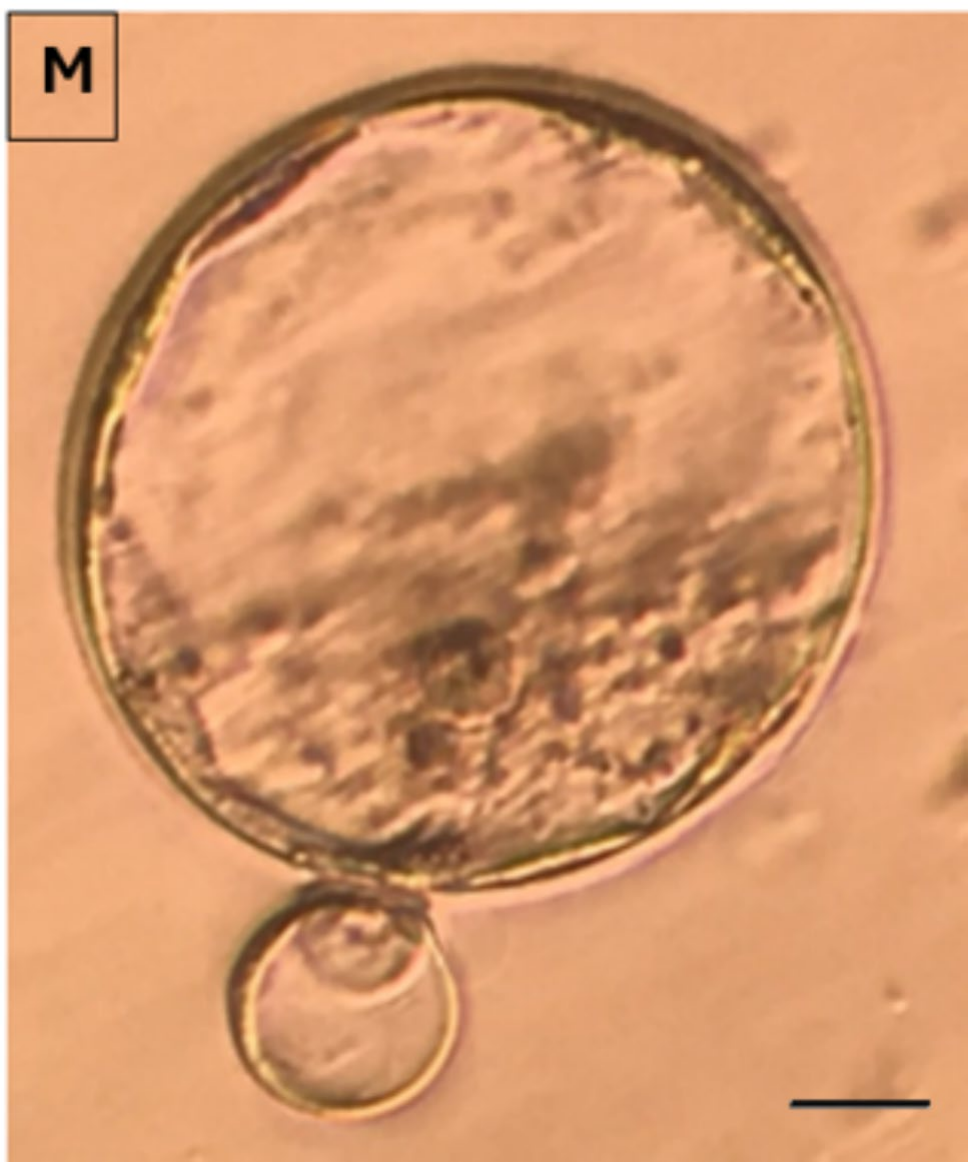
2-cell good quality (**A**) and poor quality embryo (**B**) at 24 hours; 4-cell good quality (**C**) and poor quality embryo (**D**) at 48 hours (Day 2); 8-cell good quality (**E**) and poor quality embryo (**F**) at 72 hours (Day 3) and, compacting embryos (**G**) and compact embryo (**H**) at the 96th hour (Day 4); Cavitating embryo (**I**; **J**) at 100 hours; Good quality (**K**) and poor quality blastocyst stage embryos (**L**) at 120 hours and, a hatching embryo (**M**) at 130 hours is demonstrated (X40)

Fertilized oocytes were transferred to fresh drops of culture medium (G1) covered with mineral oil. On day 2, 3, and 5, morphologic evaluation were performed. Embryos with less than 10% fragmentation and 2-4 evensized blastomeres without any vacuolization, granulation, and multinucleation on Day 2, seven to eight evensized blastomeres on day 3 and a visible intact outer cell mass with an intact, homogeneous inner cell mass on day 5 were considered as top quality embryos and blastocysts respectively and the rest as bad quality embryos and blastocyts respectively (Fig. 9). The percentage of top quality embryos were calculated as the number of top quality embryos on day 2,3,5 / the number of embryos transferred ×100.

### Statistical Analysis

SPSS statistical program (Version 22) was used to analyze the data. Data were expressed as mean ± standard deviation (SD). Differences between the groups were evaluated by t-test. All tests were interpreted with a significance level of 95% (*p* < 0.05).

## Results

The results obtained for 8 parameters among 17 indicators of ART laboratory indicators accepted by ESHRE Special Interest Group of Embryology, (Vienna 2017) and additional parameters analysing the embryo qualities on 2 nd and 3 rd day were demonstrated in Table 1 and 2 respectively.

**Table 1:** 17 indicators (8 of which our study included) accepted as ART (in vitro fertilization) performance laboratory indicators by ESHRE Special Interest Group of Embryology, Vienna 2017. MII: Metaphase 2; MI: Metaphase 1

PI (Performance indicators) and KPI (Key performance indicators)	Competency (%)	Control (97 oocyte)	APB-Tech (112 oocyte)	*p*-value
Degeneration rate (%) (Number of damaged or degenerated oocyte / Number of injected MII oocytes x100)	≤10	6	6.4	0.453
Normal fertilization rate (%) (Number of fertilized oocyte rate / Number of injected MII oocytes x100)	≥65	73.4	79.1	0.569
1 PN rate (%) (Number of 1 PN oocyte / Number of injected MII oocytes x100)	<3	1.2	1.3	0.238
Cleavage rate (%) (Number of cleaved embryo on Day 2 / Number of fertilized oocyte on Day 1 x100)	≥95	96.3	98.2	0.651
2 nd day embryo development rate (Number of 4-cell embryo on Day 2 / Number of fertilized oocyte on Day 1 x100)	≥50	78.4	81.2	0.752
3 rd day embryo development rate (Number of 8-cell embryo on Day 3 / Number of fertilized oocyte on Day 1 x100)	≥45	72.8	78.5	0.644
Good quality embryo development rate (%) (Number of good quality blastocyst number on Day 5/ Number of fertilized oocyte on Day 1x100)	≥30	39.2	46.6	0.045
Blastocyst development rate (%) (Number of blastocyst on Day 5/ Number of fertilized oocyte on Day 1 x100)	≥40	46.5	54.4	0.036
Good quality blastocyst rate (%) (Number of top-quality blastocysts on Day 5/ Number of fertilized oocyte on Day 1 x100)		11.6	23.4	0.047

**Table 2:** Clevage stage embryo qualities among groups.

Embryo quality	Control (97 oocyte)	APB-Tech (112 oocyte)	*p*-value
Day 2 (% top-quality embryos/2-PN)	29.0 ± 1.8	37.9 ± 2.0	0.047
Day 3 (% top-quality embryos/2-PN)	31.1 ± 1.9	38.5 ± 1.9	0.038

**Fig. 10:**
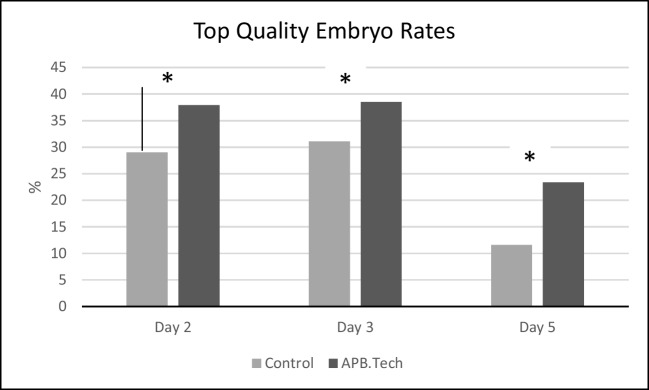
Top quality embryo rates on Day 2, 3 and 5

Good quality embryo (Total, Day 2 and Day 3) rates (*p*=0.045, 0.047, 0.038 resp), good quality blastocyst rates (*p*=0.047) and blastocyst development rates (*p*=0.036) were significantly higher in APB-Tech group (Fig. 10), while there were no difference for abnormal fertilization (1 ronucleus) rate (*p*=0.238), oocyte degeneration rate (*p*=0.453), normal fertilization rate (*p*=0.569), cleavage rates (*p*= 0.651), 2 nd and 3 rd day embryo development rates (*p*=0.752, *p*=0.644).

## Discussion

Contribution of the sperm quality on IVF success lead to a need for developing novel techniques to choose better sperm cells with the highest ‘compotency’. Routinely used sperm selection techniques in IVF labs suggest to provide a healthier ‘sperm population’ that are based only on the motility and morphology patterns of the sperm, which is reported not to reflect the overall fertility potential of sperm cells [32, 33]. Sperm DNA fragmentation, a marker of apoptosis, is reported to be significantly associated with, embryo quality [34], pregnancy and miscarriage rates [35, 36] and ICSI outcome [37]. It has been reported that, the two most commonly used sperm selection techniques (swim-up and DGS) do not decrease the rate of DNA fragmentation level of a sperm population to an acceptable level [33].

There are more advanced techniques for apoptosis detection such as electrophoresis [22] and MACS [19] which can only be used for diagnostic purposes or other techniques such as IMSI [20] and hyaluronan binding [21] which do not offer to select apoptotic sperm. Diagnostic methods include fixation, etc., which restrict the usage of the sperm for ICSI procedure. A similar method, magnetic activated cell sorter (MACS), provides a sperm suspension with a relatively reduced rate of apoptosis instead of a single sperm [19]. In addition, the effect of the magnetic field on sperm cells and the stress that sperm cells are exposed to during these processes may be detrimental [38]. All these techniques are rarely used in IVF laboratories because of high costs, and the need for extra equipment and training while none of those ensure the selection of a ‘single, and a ‘viable’ sperm cell devoid of apoptotis which ‘can’ be used for microinjection.

Recently, we introduced a new, easy and a reliable technique, APB-Tech, which enable IVF professionals to diagnose and select ‘a non-apoptotic and a viable sperm for microinjection’ via light microscopy [29]. This technique is based on the principle that Annexin-V can bind to phosphatidylserine (PS) located on the outer leaflet of the plasma membrane in apoptotic cells as an early apoptotic marker [29]. The technique can be used either for diagnostic purposes or interventional, and is user-friendly allowing users without any need for training or extra equipments. Besides, sperm cells are prevented against different kinds of extrinsic stress that can be triggered in various processes and environments.

To test the accuracy and reliability of the APB tech we compared the DNA fragmentation levels of APB-Tech with two reliable and well-known methods (SCSA, TUNEL), and found statistically similar results (13.51% for APB, 15.56% for SCSA and 17.27% for TUNEL, *p*>0.05) in our previous study [29]. We then performed sperm survival test (SST) for toxicity testing which is one of the most commonly used technique to test the toxicity of any solution, medium and consumables to be used in in vitro fertilization [39]. Sperm survival rates (total motility and motility subgroups rates) for all tested time intervals (2, 4, 6, 8, 24, 48 hours) were statistically similar with the control group proving the reliability of the technique without an adverse effect [29].

In the recent study, as the next step, we tested the technique’s effect on IVF outcome which was the main goal of the technique. The outcome of cycles in which APB-Tech is used, were compared with the results of classic cycles without the technique.

Eight parameters among ‘ART laboratory success indicators’ accepted by ESHRE Special Interest Group of Embryology, Vienna 2017, were assessed including degeneration, normal fertilization, abnormal fertilization (1PN), 2 and 3rd day embryo development, good quality embryo, blastocyst development and good quality blastocyst rates. Among those parameters we observed a significant increase in good quality embryo, good quality blastocyst and blastocyst development rates in the cycles where APB-Tech is used although other parameters were not significantly different.

According to our results, the basic effect of the technique is detected on the quality and development rates of the embryo and blastocysts. This finding supports the findings of studies which report that embryo and blastocyst quality and development rates are affected by sperm DNA fragmentation [40–42]. The referenced studies together with some others report no effect of sperm DNA on the fertilization rates verifying our results obtained for the fertilization rates [43]. Our results that embryo and blastocyst qualities and development rates are affected while fertilization rates are not, is compatible with and supports the results of the data that sperm genome is activated on the 3rd day of embryonic development [44, 45]. Since blastocyst formation occurs in 5 th day of embryonic development, it is comprehensible to observe the expected outcome on this stage. The results obtained for degeneration rates seem not to be affected by the technique as expected, but it provide evidence about its nontoxic nature during IVF procedures.

The promising results observed for these important parameters of IVF is an indicator for its potential to achieve the expected positive effects. As this study is conducted on mice, it is expected to have a much higher impact in human studies due to the genetic similarity and low DNA fragmentation rates of mice, as well as being fertile.

The developed method is expected to positively affect ICSI parameters and treatment outcome and minimize neonatal health risks by enabling professionals to select sperm that is more likely to be chosen by natural selection mechanisms.

Further studies including pregnany, ongoing pregnancy, miscarriage and live-birth rates are needed to investigate the overall effects of the technique on IVF outcome before implementing the technique on human IVF studies.

## Data Availability

The data is available upon request.
